# Daily Fluid Intake in People With Newly Diagnosed Parkinson's Disease Is Reduced Compared With Controls

**DOI:** 10.1155/padi/2440967

**Published:** 2025-10-09

**Authors:** Isobel J. Sleeman, Angus D. MacLeod, Clare Tarr, Collette McGhee, Claire Fyfe, Carrie Stewart, Karen Scott, Phyo Kyaw Myint, Alexandra M. Johnstone

**Affiliations:** ^1^School of Medicine, Medical Sciences and Nutrition, University of Aberdeen, Foresterhill, Aberdeen AB25 2ZD, UK; ^2^Speech and Language Therapy Department, Aberdeen Royal Infirmary, Foresterhill, Aberdeen AB25 2ZN, UK; ^3^Rowett Institute, School of Medicine, Medical Sciences and Nutrition, University of Aberdeen, Foresterhill, Aberdeen AB25 2ZD, UK

## Abstract

**Background:**

Parkinson's disease (PD) is an age-related neurodegenerative condition with a range of motor and nonmotor symptoms. Nonmotor symptoms such as constipation and orthostatic hypotension can occur at any stage, while dysphagia is common in later stages of the disease. Previous work by our group showed that people with PD who lose weight within a year of diagnosis had a poorer prognosis. In this study, we explored whether fluid intake was also reduced in people with newly diagnosed PD.

**Materials and Methods:**

We invited people with newly diagnosed PD (within 6 months of a diagnosis or longer if not requiring treatment) to join the study. Controls were household members of the participants with PD. Participants all underwent the same assessments, including a 24-h dietary recall, a video-recorded swallowing assessment, and grading of stool sample consistency using the Bristol Stool Chart.

**Results:**

We recruited 30 participants, 19 with PD and 11 household controls. People living with PD reported significantly lower fluid intake from drinks (control median = 1799 mL, PD median = 1124 mL, *p*=0.005 for difference in medians). People with PD drank fluid slightly slower than the controls, 6.0 mL/second vs 7.5 mL/second, but this did not reach statistical significance. Participants with PD had significantly harder stools than controls, with a mean Bristol Stool Chart number of 3.2 vs 4.6 for controls (*p*=0.01).

**Conclusion:**

PD is associated with significantly reduced intake of fluids from beverages around the time of diagnosis, which may contribute to constipation and orthostatic hypotension.

## 1. Introduction

Parkinson's disease is the second most common age-related neurodegenerative condition. Global prevalence has doubled in the past 25 years, with 8.5 million people affected worldwide in 2019 [1]. While the condition is best known for its motor symptoms—tremor, postural instability, and bradykinesia—nonmotor symptoms often significantly impact the quality of life. These include anosmia, constipation, orthostatic hypotension, and dysphagia. Dysphagia is rare at the time of diagnosis but becomes increasingly common as the disease progresses. It typically affects swallowing liquids more than solids and leads to a reduction of fluid intake, estimated at 300 mL/day [2]. At the point of clinical diagnosis with Parkinson's, however, self-reported rates of dysphagia are low, at approximately 12% [3].

The constipation and orthostatic hypotension seen in Parkinson's disease both have a neurogenic component [4, 5]. We hypothesised that reduced fluid intake might also be a risk factor or contributor to these symptoms of PD and that if so, fluid intake would be reduced when these symptoms emerge in the prodromal phase or early clinical phase of the illness. Therefore, we designed a case–control study to test this hypothesis in people with recently diagnosed PD.

## 2. Methods

This case–control study was approved by the Hampstead Ethics Committee (Reference: 22/PR/1265). Cases were recruited from local NHS Movement Disorders clinics within neurology and geriatric medicine, within 6 months of diagnosis with PD or longer if no treatment was required. We defined the time of diagnosis as the first time a probable diagnosis of PD was recorded by a hospital specialist. Controls were household members of the cases. After obtaining written informed consent, participants attended two study visits, which involved assessment of medical/medication history, questionnaires, examination using the Movement Disorder Society Unified Parkinson's Disease Rating Scale (MDS-UPDRS) [6], and a swallowing test. The questionnaires included the Swallowing Disturbance Questionnaire, which is validated in people with PD [7]. Swallowing was also directly assessed by video-recording participants drinking a 150 mL (mL) glass of water [8], with recordings watched later by two speech and language therapists blinded to the participants' diagnostic group. The cup was weighed before and after the test to determine the weight of water consumed accurately. They recorded the time taken to complete the test and the number of sips taken, and from this, they calculated drinking speed (mL/second).

Between visits, dietary intake was assessed over one 24 h period using a structured telephone interview. A nutritionist used a multiple recall pass method to elicit information about food, fluid, and supplement intake the previous day (midnight to midnight). The dietary data were then entered into the nutritional analysis software Nutritics to determine the fluid content of food and beverages consumed. Participants collected stool samples for analysis, and laboratory staff graded the samples using the Bristol Stool classification [9].

Data were analysed using appropriate parametric and nonparametric statistical tests in SPSS.

## 3. Results

We recruited 19 cases and 11 household controls. Their characteristics are presented in Table 1. Participants with PD were more likely to be male (78%) than controls (27%). On average, participants with PD (71 years) were also older than controls (67 years). For participants with PD, the median disease duration was 2.3 months (interquartile range 1.2–5.6 months), and median Hoehn and Yahr Stage 2 (interquartile Range 2-3).

Participants with PD drank a mean of 1124 mL per day, which was significantly less than household controls (1799 mL), *p* < 0.001. Participants with PD consumed 794 mL of fluid from food, which was slightly lower than controls, who consumed 905 mL, though this did not reach significance (*p*=0.19). Overall, the total fluid intake in participants with PD was 787 mL less than that of controls (*p*=0.04). Participants with PD consumed more calories than controls: 1913.7 kcal (SD 447.4) vs. 1764.7 (SD 699.4), though this did not reach significance.

Participants with PD had higher median scores on the Swallowing Disturbance Questionnaire than controls (3 vs. 0, *p*=0.03). Participants with PD drank water more slowly than controls (6.0 vs. 7.5 mL/second); however, this did not reach significance (*p*=0.23). Participants with PD had significantly lower scores on the Bristol Stool Chart for consistency, suggesting a tendency towards constipation (mean score 3.2 vs. 4.6, *p*=0.01).

## 4. Discussion

We report here that fluid intake is reduced by approximately 800 mL per day, on average, in people with Parkinson's in the early clinical phase (within 6 months from diagnosis, or untreated), compared to household controls. Although fluid intake has not been assessed in patients at this stage of the disease before, it has previously been reported that patients with an average disease duration of 9 years drank approximately 300 mL less a day than healthy controls [2]. One meta-analysis revealed that a third of community living patients with Parkinson's report symptoms of dysphagia, and that its prevalence is correlated to disease severity [10].

Self-reported dysphagia is rare in the early clinical phase; however, other factors may likely be contributing to reduced fluid intake. Our study found a small nonsignificant reduction in drinking speed, which is in line with the current literature [11]. We hypothesise that other aspects of Parkinson's may also limit fluid intake, such as tremor of the lip and arm, bradykinesia, rigidity, especially of the neck, and cognitive changes to short-term memory and motivation.

We acknowledge that our study has limitations. Chief amongst them is the small sample size: 19 participants with Parkinson's and 11 controls. The small sample size exacerbated the suboptimal participant matching: most of the Parkinson's group were male. At the same time, most controls were female, due to the higher incidence of Parkinson's in men and our use of household controls. This household matching was included to try and minimise the confounding effect of dietary differences in the study. Although the small number of participants precluded multivariate analysis to assess the impact of sex on fluid intake, we ensured that we used the appropriate nonparametric statistical tests to analyse the data appropriately, and the *p* value of 0.005 suggests that the difference was highly significant despite the small size. While dietary intake has been widely studied, most of the research has focused on the intake of food and especially energy. As such, there is no ‘gold standard' method of assessing fluid intake, which may affect the accuracy of our results. Finally, we cannot exclude the possibility that people with PD systematically underestimate fluid intake due to cognitive changes or apathy associated with Parkinson's. However, we are unaware of any research that investigates this possibility.

Despite these limitations, this study highlights a potentially interesting new insight into nonmotor symptoms in Parkinson's. International consensus suggests that older women should consume 2 L of fluid a day from food and beverages, and older men 2.5 L [12]. On this basis, participants in our study with Parkinson's were not fulfilling their daily fluid requirements, which are a risk factor for constipation, orthostatic hypotension, and delirium associated with PD [13].

Future studies are needed to (i) validate methods to quantify fluid intake in individuals with Parkinson's disease and (ii) replicate these findings in a larger cohort and determine any association with gender, or (iii) Parkinson's symptoms such as apathy, thirst sensation, and severity of motor symptoms affecting the dominant arm.

## Figures and Tables

**Table 1:** Participant characteristics.

	Parkinson's (*n* = 19)	Control (*n* = 11)	Significance
Age at first appointment (median, IQR)	71 (68–75)	67 (63–72)	*p*=0.16^a^
Male (number, %)	15 (78%)	3 (27%)	*p*=0.009^b^
Time since diagnosis (months, IQR)	2.3 (1.2–5.6)	n/a	
Hoehn and Yahr stage (median, IQR)	2 (2-3)	n/a	
MDS-UPDRS Part 3 (median, IQR)	30 (24–33)	n/a	
Daily fluid in mL from drinks (mean, SD)	1124 (375)	1799 (652)	*p*=0.005^c^
Daily fluid in mL from food (mean, SD)	794 (369)	905 (220)	*p*=0.19^a^
Daily total fluid in mL from diet (mean, SD)	1917 (479)	2704 (758)	*p*=0.04^a^
Swallowing disturbance scale score (median, IQR)	3 (2–6)	0 (0-1)	*p*=0.03^c^
Swallowing speed (mL/second) (mean, SD)	6.0 (2.7)	7.5 (3.8)	*p*=0.23^a^
Bristol stool chart (mean, SD)	3.2 (1.5)	4.6 (1.2)	*p*=0.01^a^
Calorie intake (kcal, mean, SD)	1913.7 (447.4)	1674.7 (699.4)	*p*=0.13^a^

*Note:* Movement disorder society united Parkinson's disease rating scale Part 3 (MDS-UPDRS Part 3).

Abbreviations: IQR, interquartile range; mL, millilitres; SD, standard deviation.

^a^ *t*-test.

^b^Fisher's exact test.

^c^Mann–Whitney *U* test.

## Data Availability

The data that support the findings of this study are available on request from the corresponding author. The data are not publicly available due to privacy or ethical restrictions.
